# Recurrent CSPs after Transcranial Magnetic Stimulation of Motor Cortex in Restless Legs Syndrome

**DOI:** 10.1155/2012/628949

**Published:** 2012-11-19

**Authors:** Aulikki Ahlgrén-Rimpiläinen, Hannu Lauerma, Seppo Kähkönen, Juha Markkula, Ilpo Rimpiläinen

**Affiliations:** ^1^Forensic Psychiatry, National Institute for Health and Welfare, P.O. Box 30, 00271 Helsinki, Finland; ^2^Psychiatric Hospital for Prisoners, P.O. Box 49, 20251 Turku, Finland; ^3^BioMag Laboratory, Helsinki University Central Hospital (HUCH), P.O. Box 340, 00029, Helsinki, Finland; ^4^Neuropsychiatric Outpatient Clinic and the Sleep Research Unit, Turku University Hospital, University of Turku, P.O. Box 52, 20521 Turku, Finland; ^5^Department of Clinical Neurophysiology, Helsinki University Central Hospital, P.O. Box 1020, 10601 Helsinki, Finland; ^6^Institute of Biomedical Engineering, Tampere University of Technology, 33101 Tampere, Finland

## Abstract

*Aims.* The aim of this study was to investigate the motor control and central silent period (CSP) in restless legs syndrome (RLS). *Methods.* Transcranial magnetic stimulation was focused on the dominant and nondominant hemispheric areas of motor cortex in six subjects with RLS and six controls. The responses were recorded on the contralateral abductor digiti minimi (ADM) and tibialis anterior (TA) muscles with intramuscular needle electrodes. *Results.* No significant differences were found in the motor conduction or central motor conduction time, in the latency, or in the duration of the CSPs between or within the groups, but multiple CSPs were observed in both groups. The number of the CSPs was significantly higher in both ADMs and in the dominant TA (*P* ≤ 0.01) in the RLS group compared to the controls. *Conclusion.* Descending motor pathways functioned correctly in both groups. The occurrence of the recurrent CSPs predominantly in the RLS group could be a sign of a change of function in the inhibitory control system. Further research is needed to clarify the role of the intramuscular recording technique and especially the role of the subcortical generators in the feedback regulation of the central nervous system in RLS.

## 1. Introduction

Restless legs syndrome (RLS) is characterized by an unusual, almost indescribable sensation in the lower extremities, and sometimes the arms or other body parts are involved. This irresistible urge to move the legs, or unpleasant sensations begin or worsen during periods of rest or inactivity, and the occurrence of the symptoms at rest are often partially or totally relieved by movement. The symptoms are at worst in the evening or night or only occur in the evening or night [1]. RLS is considered to be a sleep disorder as well as a sensory-motor disorder, and its prevalence approaches 3.9%–14.3% based on the minimal 4 criteria of international restless legs syndrome [1, 2] and 2.2%–7.9% when a specific severity/frequency is taken into account [3]. 

The subtype 1 is represented in individuals who experience symptoms of RLS before the age of 45 years and have a family history and a possible genetic predisposition. The subtype 2 is characterized with a later onset of symptoms that are mostly related to an iron deficiency, a pregnancy, or an end-stage renal disease [4]. RLS shares many features with a neuroleptic-induced akathisia [5]. Treatment with dopaminergic medications is, according to newly revised guidelines on management of RLS, still recommended as first-line treatment, even though there is also evidence of efficacy on other agents [6]. Mechanisms underlying the pathogenesis of RLS may be a central dopamine dysfunction, a hyperexcitability of circuitry motor neurons, and an impairment of subcortical cerebral generators. The altered function of the dopamine regulation is probably connected to changes in brain iron metabolism. There are studies showing low brain iron stores, especially in substantia nigra in RLS, and also genetic factors have been identified [4, 7].

Transcranial magnetic stimulation (TMS) allows the examination of the descending motor pathways from the motor cortex down to the target muscles. The activation of the primary motor cortex can be measured as different types of motor responses in the desired muscles. The prevailing muscle activation can also be interrupted by TMS, and an induced central silent period (CSP), inhibition, can be measured [8, 9]. 

In general, earlier TMS studies have shown that descending corticospinal motor pathways function correctly in RLS, but there are several studies showing inconsistent results concerning inhibitory control systems, especially the central silent period. In one of the earliest studies, intracortical inhibition (ICI) was found decreased in upper and lower limbs, whereas intracortical facilitation (ICF) was decreased in the lower limbs referring to changes in the corticosubcortical motor excitability in RLS [10]. Some TMS studies have reported no significant differences on the central inhibition between subjects with RLS and controls, but at the same time they reported reduced intracortical inhibition correlating to the body side affected by sensory-motor symptoms in RLS, involving especially arms [11]. Some studies have reported a shorter [12–15] or even increased CSP duration [16] in RLS compared to controls. After treatment with dopamine agonists the central silent period has been found lengthened [15] or unchanged [16], but there are reports of CSP being shortened in the upper and prolonged in the lower limbs [13, 17]. Circadian variability has also been demonstrated in studies with medicated subjects and unmedicated subjects with RLS, and the CSP has shown diminishing tendency in subjects with RLS at night compared to controls [18]. Periodic limb movement disorder is a distinct disorder although it often occurs simultaneously with RLS. Approximately 80% of individuals with RLS have periodic limb movements (PLMs) during sleep [13].

Because earlier studies have demonstrated inconsistent results especially in the duration of the CSPs, we chose a different kind of technique to investigate the motor control, especially the silent period, in a small population with RLS compared to healthy controls. We also aimed to find out possible side-to-side differences within the RLS group. We used a combination of intramuscular recording electrodes and a single pulse TMS to investigate the dominant and nondominant motor cortex areas of abductor digiti minimi (ADM) and tibialis anterior muscles (TA). 

## 2. Materials and Methods

The subjects with RLS were recruited from university and community hospitals in Turku. To rule out other primary sleep disorders or any secondary causes the subjects with RLS underwent several medical specialist examinations including neurological and psychiatric examinations before participating in our study. Blood tests for renal and hepatic function, blood glucose, ferritin, B-12 vitamin, blood cell account, and plasma level of hemoglobin and, if necessary, electroencephalography, sleep polysomnography as well as brain computerized tomography were also checked prior to the investigations. Subjects identified having a periodic limb disorder (PLMD) or Parkinson's disease were not recruited in our study.

A total number of 6 subjects with an ICD-10 diagnosis of RLS (3 females, 3 males, mean age = 60.3, standard deviation (sd) = 10.3) and 6 healthy volunteers of the hospital ward personnel as controls (3 females, 3 males, mean age = 41.2 years, sd = 5.9) with no history of severe illnesses or traumatic injuries of the extremities or the head were included in the study. The participants with RLS fulfilled the essential diagnostic criteria for RLS [1, 2]. They were identified having the RLS subtype 1: the duration of RLS had been several years, and the symptoms had become more evident and worse each subsequent year. They described a positive family anamnesis for RLS and also symptoms in their arms. All the study participants were without any central nervous system (CNS) affecting drugs or dopaminergic medication at least for seven days prior to the investigation. The right handedness of the participants (i.e., RLS and control) was checked with the help of the Edinburgh Handedness Inventory [19]. All the subjects were informed about the study, and they gave their written informed consent prior to the study. The clinical interviews and investigations were performed at the Psychiatric Community Hospital of Turku, Finland, (volunteers with RLS) and at the Laboratory of Clinical Neurophysiology and Psychiatry, Ekåsen Mental Hospital, Ekenäs, Finland, (controls). The study was approved by the local ethics committee.

TMS was performed by using a commercially available magnetic stimulator, Cadwell MES-10, supplied with a round coil that had an external diameter of 9 cm. The stimulation intensity constantly exceeded the motor threshold level. A biphasic stimulation pulse with an intensity of 60 to 80% of the maximum capacity of the device was applied. In each series of stimuli, altogether five repetitive stimuli were given with a time interval of 1 to 5 seconds. For the stimulation of abductor digiti minimi muscles (ADM) in the upper extremities, the center of the coil was placed at the midpoint between the upper tip of the earlobe and the vertex, corresponding the temporal motor area, contralateral to the recording side of the responses. The most optimal site for the coil to stimulate tibialis anterior muscles (TA) in the lower extremities was to have the center located in the central area close to the vertex. Adjustments of the coil positions were made to achieve the most favorable site of the stimuli of respective muscles. The shaft of the coil was directed backwards. 

The responses were recorded with the help of a Dantec KeyPoint device and a pair of monopolar needle electrodes that were inserted into the ADM and TA muscles at a distance of 3 cm from each other. The cathode was positioned proximal to the anode. The intramuscular recording technique with needle electrodes provides a possibility to measure a high number of single motor units simultaneously, and it aids the measurement of the high-frequency components of the muscular activity. In maximum voluntary muscle activation the method demonstrates practically total activation of the corticospinal and lower motor neuron tracts reaching to that definitive muscle. The maximum muscle motor unit recruitment can be visually verified as a comprehensive muscle interference pattern [20]. This method gives a more precise picture of the suppression of the high-frequency components in the muscle compared to the surface electrode measurement that provides information from the motor activity compound of the muscle [21–23]. 

Recommendations for the optimal TMS technique, intensity, and muscle contraction were applied [24]. The optimal stimulus location was determined by mapping the primary motor area with the stimulating round coil until the best response according to amplitude criteria was achieved in the target muscle by a constant stimulus intensity that was above the level of the motor threshold. TMS was performed on the motor cortex of the dominant and the nondominant hemispheric areas. 

To measure the motor distal latency (MDL) and the latency of F responses (F), respectively, electrical stimuli rectangular pulses with duration of 0.2 ms and intensities of 10 to 50 mA were given at the ulnar and the peroneal nerves at the wrist and at the fibular head on the lateral side of the knee, respectively. The stimulation intensity for both MDL and F response was determined by gradually increasing the electrical current until a supramaximal motor response was achieved. For a measurement of MDL, a time frame of 25 ms was applied, and the latency was measured from the onset of a single supramaximal motor response. To measure F waves, 10–20 stimuli were applied with a time interval of 1-2 sec, and the F waves were identified. Time frames of 80–100 ms were applied, and F was calculated from the minimum latency of the responses (Table 3). The target muscles were relaxed, and the electromyographic inactivity was controlled by the recording electrodes. 

For the analysis of muscle activation, the following parameters were recorded: (1) MDL = respective motor distal latency to ulnar nerve stimulation at wrist and to peroneal nerve stimulation at fibular head, (2) latency of F response to ulnar nerve stimulation at wrist (F_u_) and to peroneal nerve stimulation at fibular head (F_p_), MDL is excluded from the F latency, (3) motor conduction time from the cortex to ADM (MCTa) and to TA (MCTt). Based on the previous recordings the following parameters were calculated: (4) central motor conduction time from motor cortex to neck: CMCT_n_ = MCTa − (F_u_/2 + MDL) and (5) Central motor conduction time from motor cortex to lumbar area: CMCT_l_ = MCTt − (F_p_/2 + MDL). 

For the analysis of the CSP in each voluntarily maximally preactivated muscle, the following parameters were recorded on the contralateral side to a series of five magnetic stimuli. The presence of the CSP was defined as a simultaneous decrease of amplitude of muscular activity below 0.05 mV/division in five consecutive measurements. The level was judged by visual inspection. The onset of the CSP was defined as a start point of a simultaneous lowering of muscular activity below the defined level in five consecutive responses and the end of it as the amplitude of the activity exceeded the level. The duration of the CSP was calculated in milliseconds between the onset and end of CSP. Maximum preactivation of the muscle activity was defined as a full electromyographic interference pattern in a time frame of 500 ms and a sensitivity of 1 mV/division. The parameters were applied to measurement of inhibitory responses, too. Stimulation intensity was the same used to elicit motor evoked potentials (MEPs). Responses disturbed by artifacts due to movements, external disturbances, or an insufficient preactivation of the motor activity were excluded, and the test was repeated. 

For statistical analyses, PASW for Windows 18 was used. Groups (i.e., restless legs syndrome and control) were compared with the help of the Mann Whitney test for independent samples. The comparison for the number of the CSPs was performed with the help of the Chi-square test. The Wilcoxon signed rank test for related samples was applied to analyze results between the dominant and nondominant body sides within the groups (i.e., RLS and controls) 

## 3. Results and Discussion

### 3.1. Results between the Groups (i.e., RLS and Controls)

#### 3.1.1. Mann Whitney Test and Chi-Square Test

**Figure 1:**
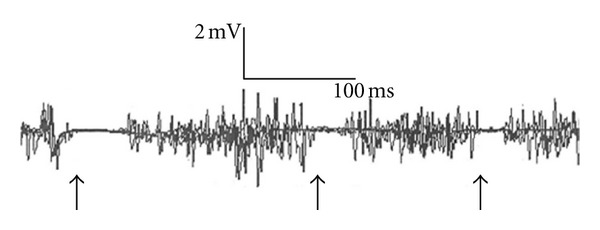
Example of multiple recurrent CSPs observed in subjects with RLS.  Figure 1 demonstrates an inhibitory response of a subject with RLS. After TMS on the nondominant hemisphere of this RLS subject 3 separate CSPs in the nondominant ADM (arrows pointing to the suppression of muscle activity) could be observed.

All subjects completed the study protocol. Tables 1, 2, and 3 summarize the obtained results (mean values and standard deviations). No significant differences were obtained between the RLS and control groups in MCT, in CMCT (Table 1), or in MDL (Table 2). Interestingly, after almost each TMS impulse, up to one to three separated compounds of CSP in the target muscles were observed.  Figure 1 presents an inhibitory response of a participant with RLS after TMS on the nondominant motor cortex area of ADM demonstrating three separate periods of CSPs. 50% of the total six participants with RLS demonstrated two compounds of CSPs in both ADMs, and even a third CSP could be detected in both ADMs in one participant with RLS. Two RLS participants demonstrated two CSPs in both TAs. One participant with RLS demonstrated even a third CSP in the nondominant TA. Only one control person had two CSPs in the dominant TA, and only two controls had two CSPs in the nondominant TA. 

The RLS and control groups did not differ significantly on the latency and on the duration of the first CSP compound or on the latency and the duration of the second compounds of the CSPs in TAs (Table 3). We summarized the durations and the number of all CSP compounds in each site of the stimulation after each TMS impulse, that is what we call a total duration or a total number of the CSPs. No significant differences were observed in the total duration of the CSPs (ADMs: *U* ≥ 14, *P* > 0.59; TAs: *U* = 11.0, *P* = 0.31) between the groups.

The RLS group showed a significantly higher number of CSPs in the dominant ADM (mean value = 1.5, sd = 1.1; Chi-square test: *x* ^2^ = 11.3, *P* = 0.01), in the dominant TA (mean value = 1.3, sd = 1; Chi-square test: *x* ^2^ = 11.3, *P* = 0.01), and in the nondominant ADM (mean value = 1.7, sd = 0.8; Chi-square test: *x* ^2^ = 9.5, *P* = 0.009) compared to the controls. The Chi-square test showed no significant differences for the number of the CSPs in the nondominant TA (RLS: mean value 1.5, sd = 0.6; controls: mean value = 1.3, sd = 0.5, *x* ^2^ = 0.33, *P* = 0.56) (Table 3).

### 3.2. Results within the Groups (i.e., RLS and Controls) between the Dominant and the Nondominant Body Sides (Wilcoxon Test)

No side-to-side differences were observed in MCT, CMCT, or MDL (Tables 1 and 2), in the latency, in the duration, or in the total duration of the CSPs (Table 3) within the groups. The number of the CSPs did not differ significantly within the RLS group (ADM: *Z* = −0.45, *P* = 0.66; TA: *Z* = −0.45, *P* = 0.66) nor within the control group (ADM: *Z* = 0.0, *P* = 1; TA: *Z* = −0.58, *P* = 0.56) (Table 3) between the body sides.

### 3.3. Discussion

Relevantly to earlier studies, the corticospinal motor pathways functioned correctly in subjects with RLS compared to controls. However, we found that the central inhibition consisted of up to one to three separate compounds of CSPs. The number of the CSPs was significantly higher in both ADMs and in the dominant TA in the participants with RLS compared to the controls. The separate compounds of the CSPs did not differ significantly on the duration or on the latency between the RLS group and the control group. No significant side-to-side differences were observed within the groups. The recurrent CSPs showed a diminishing duration (Table 3).

In literature, the early part of the CSP is described to reflect a suppression of the spinal motor nuclear activity mediated directly from the motor cortex, whereas the later part of the CSP reflects a suppression of the muscle activity by supraspinal structures presynaptic to fast descending motor pathways [25]. We used an intramuscular pair of monopolar electrodes, a technique that enables the measurement of high-frequency components of the electrical activity in the muscle, and that technique may provide additional information from the interneuronal motor control systems in the subcortical brain structures. Compared to the surface electrode recording technique, the changes in the muscular electrical activity, including the firing frequency, can be more exactly determined in the intramuscular recording [20, 22, 23], which in turn makes it easier to determine the onset and end of the silent period. Thus, the recording technique might explain the easier detection of the multiple CSPs including the first suppression and the later upcoming suppressions of muscular activity too. 

Earlier studies have reported that in healthy persons CSPs are less excitable in the dominant motor cortex and that right handers show more inhibition and less facilitation than left handers [26, 27]. Thus, these findings might also support the asymmetric results in our study the more pronounced occurrence of the recurrent CSPs on the dominant body side in RLS. The inhibitory, but not facilitatory, circuits of the hand and foot motor cortex are connected to each other [10, 11]. This might explain our study findings, which indicated that the motor control system was affected in the upper as well as in the lower extremities in RLS. The well-known fact that arms are also affected in the chronic forms of RLS (subtype 1) [28] could be supported by our study results showing recurrent inhibitions in both ADMs. 

Dopaminergic pathways are likely to be involved in the pathophysiology of RLS, and the symptoms can be markedly alleviated by dopaminergic agonists [16]. RLS as well as Parkinson's disease is supposed to have their pathophysiological origin in the basal ganglia that are to a great extent responsible for the motor control mechanisms [29, 30]. However, many genetic, pathological, and imaging data suggest that there is no direct relationship between these two disorders [31, 32]. There are studies reporting both decreased CSP [12] and increased CSP [16] in RLS, and modified, also disrupted, CSP in Parkinson's disease [33–35]. The pathophysiological basis of multiple CSPs in RLS could be located in the extrapyramidal tracks and might reflect disturbances in their neurotransmitter systems, especially a disorder in dopamine system that might promote motor symptoms in RLS by disturbing the inhibitory motor control [30]. 

Limitations of this study were the following three facts. The day time is not an optimal time to investigate RLS symptoms. The age difference was notified, but it did not play such a significant role because the symptoms of RLS were practically identical in all the participants with RLS included in the study (chronic course, severity of experienced symptoms, subtype 1, and symptoms in the arms). Because of the intramuscular recording methods, the study sample was initially planned to be small. 

## 4. Conclusions

The recurrent CSPs, in other words the increased number of separate compounds of CSPs found in our study, may reflect a dysfunction of the central inhibition process in RLS. The predominant incidence of the multiple CSPs on the dominant body side in our study could be explained by an asymmetric organization of central inhibitory control system that may be more prone to disruptions on the dominant hemisphere. These disruptions might be due to altered excitatory recovery of the motor interneuron activity and may further lead to a change in motor processing in terms of recurrent inhibitions predominantly on the dominant body side. 

Further research of eventually altered feedback system of CNS is needed, especially about the role of the basal ganglia controlling the motor cortex output and the possible asymmetric organization of the inhibitory control system. A longitudinal TMS study should be carried out in a larger RLS population, targeting various arm and leg muscle groups, using surface and intramuscular electrodes, and performing the clinical investigations preferably in bed time. 

## Figures and Tables

**Table 1:** Motor conduction time (MCT) and central motor conduction time (CMCT) in abductor digiti minimi (ADM) and tibialis anterior (TA) muscles did not differ between the subjects with RLS and controls. The results were measured on the contralateral body side after transcranial magnetic stimulation (TMS) on the dominant (Dom) and nondominant (ND) motor cortex areas.

Side of S.	*n *	MCT (ms)	MCT (ms)	CMCT (ms)	CMCT (ms)
		mean (sd)	mean (sd)	mean (sd)	mean (sd)
		Dom	ND	Dom	ND
Controls/ADM	(6)	21.2 (1.34)	21.05 (1.09)	6.53 (1.73)	6.18 (1.05)
RLS/ADM	(6)	20.75 (0.65)	21.37 (1.04)	6.82 (0.39)	7.47 (0.63)
Controls/TA	(6)	29.23 (2.30)	28.62 (1.57)	11.93 (1.56)	10.99 (1.17)
RLS/TA	(6)	28.13 (2.51)	28.15 (3.01)	11.70 (2.91)	11.27 (3.21)

Side of S.: side of hemispheric stimulation, ms: millisecond, sd: standard deviation, *n*: number of subjects.

**Table 2:** F-wave (F) and motor distal latency (MDL) measured in the dominant (Dom) and non-dominant (ND) abductor digiti minimi (ADM) and tibialis anterior (TA) muscles did not differ significantly between subjects with RLS and controls (CTR).

Side of S.	*n *	F (ms)	F (ms)	MDL (ms)	MDL (ms)
		mean (sd)	mean (sd)	mean (sd)	mean (sd)
		Dom	ND	Dom	ND
Controls/ADM	(6)	24.12 (4.14)	24.67 (2.89)	2.68 (0.4)	2.53 (0.2)
RLS/ADM	(6)	23.43 (1.27)	23.47 (1.76)	2.22 (0.25)	2.17 (0.3)
Controls/TA	(6)	26.15 (1.48)	26.72 (2.11)	4.23 (0.56)	4.27 (0.62)
RLS/TA	(6)	23.67 (1.68)	24.07 (1.84)	4.60 (0.6)	4.85 (0.44)

ms: milliseconds, sd: standard deviation.

**Table:** (a)

Duration	*n *	1.CSP (ms)	2.CSP (ms)	3.CSP (ms)	Total Dur (ms)
DOM. side of S.			mean (sd)		
Controls/ADM	(6)	67.3 (32.3)	0	0	67.3 (32.3)
RLS/ADM	(6)	44.8 (32.3)	16.7 (22.1)	10.0 (1 abs.)	63.2 (44.1)
ND. side of S.					
Controls/ADM	(6)	93.3 (64.7)	0	0	93.3 (64.7)
RLS/ADM	(6)	58.5 (39.5)	12.7 (15.0)	10.0 (1 abs.)	72.8 (48.0)

DOM. side of S.			mean (sd)		
Controls/TA	(6)	70.7 (56.0)	4.0 (9.8)	0	74.7 (65.1)
RLS/TA	(6)	36.2 (33.2)	5.0 (8.9)	15.0 (1 abs.)	43.7 (46.3)
ND. side of S.					
Controls/TA	(6)	42.7 (45.3)	7.5 (11.)	0	50.2 (48.3)
RLS/TA	(6)	44.7 (45.3)	12.3 (20.1)	0	57.0 (65.0)

**Table:** (b)

Latency	*n *	1.CSP (ms)	2.CSP (ms)	3.CSP (ms)	Total no. of CSPs
DOM. side of S.			mean (sd)		
Controls/ADM	(6)	54.7 (4.8)	0	0	1.0 (0.4) 6 abs.
RLS/ADM	(6)	44.7 (22.3)	225 (141.1)	346 (1 abs.)	1.5 (1.1) 9 abs.*
ND. side of S.					
Controls/ADM	(6)	55.5 (7.8)	0	0	1.2 (0.4) 7 abs.
RLS/ADM	(6)	53.0 (4.6)	251 (128.4)	420 (1 abs.)	1.7 (0.8) 10 abs.**

Duration		1.CSP (ms)	2.CSP(ms)	3.CSP(ms)	Total Dur (ms)

DOM. side of S.			mean (sd)		
Controls/TA	(6)	67.0 (7.8)	231 (1 abs.)	0	1.2 (0.4) 7 abs.
RLS/TA	(6)	64.2 (13.2)	270.5 (102.5)	327 (1 abs.)	1.3 (1.0) 8 abs.*
ND. side of S.					
Controls/TA	(6)	59.8 (12.3)	223.5 (127.7)	0	1.3 (0.5) 8 abs.
RLS/TA	(6)	64.7 (13.0)	125.5 (147)	0	1.5 (0.6) 9 abs.

Side of S.: side of stimulation, CSP: central silent period, ms: millisecond, DOM: dominant body side, ND: nondominant body side, sd: standard deviation, CSP: central silent period, abs.: absolute number or value of the measurement. (*P = 0.01, **P = 0.009).
